# Increased Frequency of Myeloid-Derived Suppressor Cells in Myasthenia Gravis After Immunotherapy

**DOI:** 10.3389/fneur.2022.902384

**Published:** 2022-06-29

**Authors:** Yan Wang, Chong Yan, Caixia Su, Ying Wang, Sushan Luo, Jun Lu, Chongbo Zhao, Gan Zhao, Jianying Xi

**Affiliations:** ^1^Central Lab, Huashan Hospital, Fudan University, Shanghai, China; ^2^Department of Neurology, Huashan Hospital, Fudan University, Shanghai, China; ^3^Huashan Rare Disease Center, Shanghai, China; ^4^China Key Laboratory of Medical Molecular Virology (MOE/NHC/CAMS), School of Basic Medical Sciences, Shanghai Medical College, Fudan University, Shanghai, China; ^5^Department of Pharmacy, Huashan Hospital, Fudan University, Shanghai, China

**Keywords:** myeloid derived suppressor cell (MDSC), myasthenia gravis (MG), Arginase-1, interferon γ (IFN- γ), immunoregulatory

## Abstract

Myeloid-derived suppressor cells (MDSCs) are a population of myeloid progenitor cells with immunoregulatory functions and their role in myasthenia gravis (MG) was unknown. In this study, we investigated the phenotypic and functional alterations of MDSCs in MG before and after immunotherapy. The frequency of MDSCs significantly increased and negatively correlated to that of Th1 or Th17 cells after immunotherapy. MDSCs from untreated patients with MG showed an impaired suppression of IFN-γ production in T-cells and improved immunosuppressive function was identified after immunotherapy. The MFI of Arg-1 in MDSCs also increased after immunotherapy. These findings suggested the functional difference in MDSCs before and after immunotherapy, and MDSCs might play a role in disease remission.

## Introduction

Myasthenia gravis (MG), characterized by T-cell involvement and complement dependence, is a group of autoimmune diseases manifested as fluctuating muscle weakness, typically the best in the morning and more pronounced at the end of the day (1). It is caused by antibodies against the components of the post-synaptic muscle endplate at the neuromuscular junction. Eighty percent of patients with MG have detectable antibodies against muscle nicotinic acetylcholine receptors (AChR) and a minority have autoantibody against muscle-specific tyrosine kinase (MuSK) or low-density lipoprotein receptor-related protein 4 (LRP4) (2).

Immune regulatory cells, a small set of immune cells, play an essential role in curbing excessive immune activation and maintaining immune homeostasis. Various immune cells have been found to possess a regulatory capacity, mainly including regulatory T-cells (Tregs), regulatory B-cells (Bregs), macrophages, myeloid-derived suppressor cells (MDSCs), dendritic cells (DCs), and mesenchymal stromal cells (MSCs) (3). Autoimmune disorders may arise when there are defects in the number and function of those cells. In MG, decreased frequency of CD45RA^+^ FOXP3^+^ Tregs and reduced suppressive activity of CD4^+^ CD25^high^ CD127^low^ Tregs have been found (4, 5). Unlike Tregs, Bregs are not well defined due to lacking a specific transcription factor. Both CD1d^+^CD5^+^ and CD24^high^ CD38^high^ B cells, which were described to contain an enriched population of Bregs, showed impaired production of IL-10 and suppression of T-helper polarization in MG (6).

Myeloid-derived suppressor cells are a morphologically and functionally heterogeneous population of myeloid progenitor cells with immunoregulatory functions. In humans, MDSCs were identified by HLA-DR^−−/low^ CD11b^+^CD33^+^ (HLA-DR, human leukocyte antigen–D-related) (7). Human MDSCs comprise two major subsets classified as CD33^+^CD11b^+^ HLA-DR^−/low^ CD14^+^CD15^−^ monocytic MDSCs (mMDSCs) and CD33^+^ CD11b^+^HLA-DR^−/low^CD14^−^CD15^+^ granulocytic MDSCs (gMDSCs) (8). MDSCs suppress immune responses by several distinct mechanisms, including (1) production of Arginase-1 (Arg-1), inducible nitric oxide synthase (iNOS), and reactive oxygen species (ROS) to suppress T-cell responses and induce T-cell apoptosis; (7) (2) facilitating Tregs induction by releasing IL-10 and transforming growth factor–β (TGF-β) (7, 9); and (3) impairing T-cell function by expressing PD-1/PD-L1 and indoleamine 2,3-dioxygenase (IDO) (10, 11).

Since MDSCs are posited to play a deleterious role in malignancies, they may be functionally beneficial and protective in autoimmunity (12). However, the role of MDSCs in patients with autoimmune diseases is still controversial, and few studies have characterized the role of MDSCs in MG. We hypothesized that MDSCs might have a quantitative or functional defect in patients with MG. In this study, we analyzed the frequencies of circulating MDSCs, their inhibiting capacity on IFN-γ production in T-cells, and their expression of Arg-1 in patients with MG before and after immunotherapy, trying to find the functional alterations of MDSC in MG during different disease stages.

## Methods

### Patients and Healthy Controls

Generalized MG patients with anti-AChR antibodies and healthy controls (HC) were recruited at Neurology Department, Huashan Hospital, Fudan University from 2019 to 2020. Patients who had other autoimmune diseases and received immunotherapy before enrollment were excluded. All patients and HCs in this study had no malignancies, acute or chronic infections, or other autoimmune diseases. All the patients were followed for 6–12 months after steroid immunotherapy with or without immunosuppressants. The study was approved by the Institutional Review Board of Huashan Hospital, Fudan University, and written informed consent was granted from each participant.

Clinical severity, including Myasthenia Gravis Foundation of America (MGFA) classification, quantitative MG (QMG) score (13), and Myasthenia Gravis Activities of Daily Living (MG-ADL) (14) was evaluated at baseline and 6–12 months after immunotherapy, respectively. At the same time, peripheral blood samples were collected and serum anti-AChR antibody level (cutoff:0.45 nmol/L) was detected by radioimmunoassay (Kindstar Global, China).

### Cell Isolation and Flow Cytometric Analysis

Peripheral blood mononuclear cells (PBMCs) from healthy controls and patients were freshly isolated by Ficoll-Paque Plus gradient centrifugation (Amersham Biosciences, Uppsala, Sweden).

For analysis of MDSCs, Bregs, and Treg, PBMCs were stained with the following mAbs: anti-human CD14 PE-Cy7, anti-human CD33 PE, anti-human CD11b FITC, anti-human HLA-DR PerCP-Cy5.5, anti-human CD15-APC, anti-human CD127 APC (BD Biosciences, USA), anti-human CD19 PE-Cy7, anti-human CD5 FITC, anti-human CD1d PE, anti-human CD24-PE, anti-human CD38-APC, anti-human CD3 FITC, anti-human CD4 PerCP-Cy5.5, and anti-human CD25-PE (eBioscience, USA).

For analysis of Th1 and Th17 cells, PBMCs were stimulated with 50 ng/ml PMA (Sigma-Aldrich, USA) and 1 μg/ml ionomycin (Sigma-Aldrich) for 6 h. GolgiStop (BD Biosciences) was added 1 h after stimulation. After the washing step, the stimulated cells were stained with anti-human CD4 APC-H7, anti-human CD3 PerCp-Cy5.5, and anti-human CD8 FITC mAbs (BD Biosciences, USA), permeabilized using a Cytofix/Cytoperm Plus kit (BD Biosciences) and intracellularly stained with anti-human IFN-γ PE and anti-human IL-17 APC mAbs (BD Biosciences, USA).

For intracellular staining of human Arg-1, PBMCs were cryopreserved and thawed according to a standardized protocol. Cells then were stained with anti-human CD14, anti-human CD33, anti-human CD11b, anti-human HLA-DR, anti-human CD19, and anti-human CD3 mentioned above as well as anti-human CD56-APC, anti-human CD16-PE (eBioscience, USA), permeabilized using Cytofix/Cytoperm Plus kit (BD Biosciences, USA), and intracellular stained with anti-human Arg1-Alexa Fluor 405 (R&D, USA). Then the mean fluorescence intensities (MFI) of Arg-1 in MDSCs, NK cells, monocytes, T and B-cells were detected.

Flow cytometry data were obtained using a flow cytometer (BD FACS Canto and BD FACS Aria II, USA) and analyzed with Flowjo (Tree Star, USA).

### MDSCs Depletion and Functional Assays

For the function assay associated with MDSC, inhibition of T-cells is the gold standard. Non-specific inhibition of anti-CD3/CD28 induced IFN-γ production in T-cells by intracellular staining is one of the most used methods (8). Considering the difficulty obtaining sufficient MDSCs from PBMCs in patients with MG, we evaluated the impact of MDSCs on IFN-γ production in T-cells using MDSC depleted PBMCs by flow cytometry sorting.

First, MDSCs were depleted by FACS Aria II cell sorter (Becton Dickinson, Franklin Lakes, USA) based on their expression of HLA-DR, CD11b, and CD33 (BD Biosciences, USA). Then, control PBMCs and MDSC-depleted PBMCs were stimulated with.5 μg/ml purified plate-bound anti-CD3 (BD Biosciences, USA) mAb and soluble anti-CD28 (BD Biosciences, USA) mAb for 3 days in 96-well plates (Nunc, Germany), the staining for CD3 and intracellular IFN-γ detection were performed as described above.

### Quantitative Real-Time PCR (qPCR)

Total RNA was extracted from isolated PBMC by TRIzol (Invitrogen, USA) and reverse-transcribed into complementary DNA (cDNA) using a PrimerScript RT Reagent Kit (Takara, China). Relative expressions of Arg-1, iNOS, IL-10, PD-L1, p47phox, gp91phox, and IDO were determined by normalizing the expression of each target gene to β-actin (11). Gene-specific primers are listed in Table S1. 2^−(ΔΔCt)^ was used to calculate the relative fold gene expression in samples after immunotherapy.

### Statistical Analysis

Paired (before and after immunotherapy). and unpaired (HCs and patients with MG) samples were tested for statistical significance by the non-parametric Mann-Whitney U-test and parametric Student's *t*-test, respectively. The correlation coefficient was calculated using Spearman's test for non-parametric data. Numeric values are expressed as mean ± standard deviation, and *p* < 0.05 was considered statistically significant. All statistical analyses were performed using GraphPad 8 (La Jolla, USA).

## Results

### Patients

We enrolled 30 generalized MG patients with anti-AChR antibodies and 30 HCs. The clinical characteristics of all patients and HCs are summarized in Table 1. The MGFA classification, QMG score, MG-ADL, and the titer of AChR antibody before and after immunotherapy and medication use are shown in Table S2. All the patients were given oral steroids with an initial dose of 20 mg of prednisone every day. The dose was increased by increments of 10 mg every 2 weeks without exceeding.75 mg/kg/d of body weight. The dose was maintained for 1–2 months when showing a ≥2-point reduction in ADL-MG and then reduced by 5 mg every 1–2 months. Intravenous methylprednisolone was administrated in two patients during hospitalization. Non-steroid immunosuppressants were added for patients who did not respond to steroids or refused to take a higher dose of oral steroids. All patients showed clinical improvement, 29/30 patients showed a ≥2-point reduction from baseline in MG-ADL, and only 1/30 showed a 1-point reduction.

**Table 1:** Summary of healthy controls and clinical features of 30 patients with MG.

**Features**		**HC, *n*(%)** ***n* =3 0**	**MG**, ***n*****(%)** ***n*** **=** **30**		***P*-value**
Sex	Male	17 (56.7)	14 (46.7)		0.61^#^
Age (years)		44.6 ± 12.4	49.1 ± 19.7		0.30^#^
Disease duration (months)		/	15.2 ± 36.4		
With thymoma		/	7 (23.3)		
Disease severity		/	Pre-treat	Post-treat	
MGFA classification	MMS	/	0	5 (16.7%)	0.002*
	I	/	0	2 (6.8%)	
	II	/	16 (53.3%)	21 (70.0%)	
	III	/	11 (36.7%)	2 (6.8%)	
	IV	/	3 (10.0%)	0	
QMG		/	12.8 ± 5.1	5.6 ± 3.5	<0.001*
MG-ADL		/	7.5 ± 4.0	1.9 ± 1.8	<0.001*
AChR antibody (nmol/L) (*n* = 23)		/	11.1 ± 4.8	7.9 ± 5.3	<0.001*

HCs, healthy controls; MG, myasthenia gravis; Pre-treated, before immunotherapy; Post-treated, after immunotherapy; MGFA, Myasthenia Gravis Foundation of America; MMS, minimal manifestation status; QMG score, quantitative MG score; MG-ADL, MG Activities of Daily Living; AChR, acetylcholine receptor; ^#^comparison between patients and HC; *comparison between pre-treated and post-treated patients.

### Alternation of Immune Regulatory Cells After Immunotherapy

**Figure 1:**
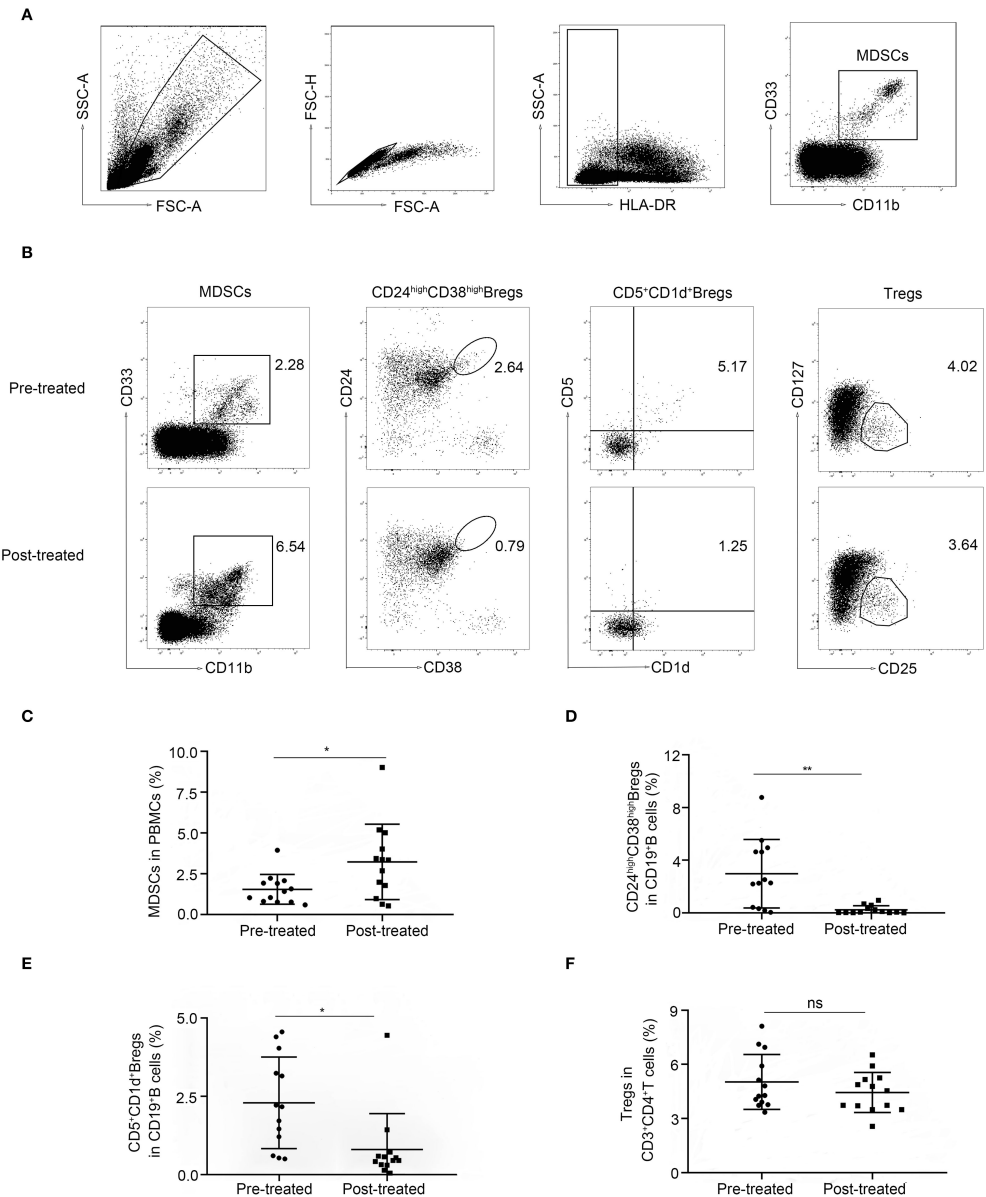
Alternation of immune regulatory cells after immunotherapy. **(A)** Sequential gating strategy for myeloid-derived suppressor cells (MDSCs) identification (HLA-DR^−/low^CD33^+^CD11b^+^) from peripheral blood mononuclear cell (PBMCs). **(B)** Representative data plots of MDSCs, Bregs, and Tregs from patients with MG before (Pre-treated) and after immunotherapy (post-treated). The frequency of MDSCs in PBMCs increased **(C)**, the frequencies of CD19^+^CD24^high^ CD38^high^ Bregs **(D)** and CD19^+^CD5^+^CD1d^+^ Bregs **(E)** in CD19^+^ B-cells decreased, while the frequency of CD4^+^CD25^+^CD27^low^ Tregs in CD3^+^CD4^+^ T-cells **(F)** showed no significant change. Horizontal lines and error bars represent mean ± SD, *n* = 13, *p < 0.05, **p < 0.01.

To investigate the dynamic change of immune regulatory cells in patients with MG, we compared the frequencies of Tregs and Bregs as well as MDSCs by flow cytometry in patients with MG (*n* = 13) before (pre-treated) and after immunotherapy (post-treated). We observed a significantly higher frequency of HLA-DR^−/low^ CD11b^+^CD33^+^MDSCs in PBMCs in patients after immunotherapy compared to baseline (*p* = 0.021. Figures 1A–C). While the frequencies of CD24 high CD38 high and CD1d^+^CD5^+^ Bregs in CD19^+^ lymphocytes decreased (*p* = 0.0032 and 0.019, respectively, Figures 1B,D,E). No significant change was observed (*p* = 0.24) in the frequency of CD25^+^CD127^low^ Tregs in CD3^+^CD4^+^ lymphocytes (Figures 1B, 1F). So, we focused on the phenotypic and functional changes of MDSCs before and after immunotherapy.

### Expansion of MDSCs in Patients With MG After Immunotherapy

**Figure 2:**
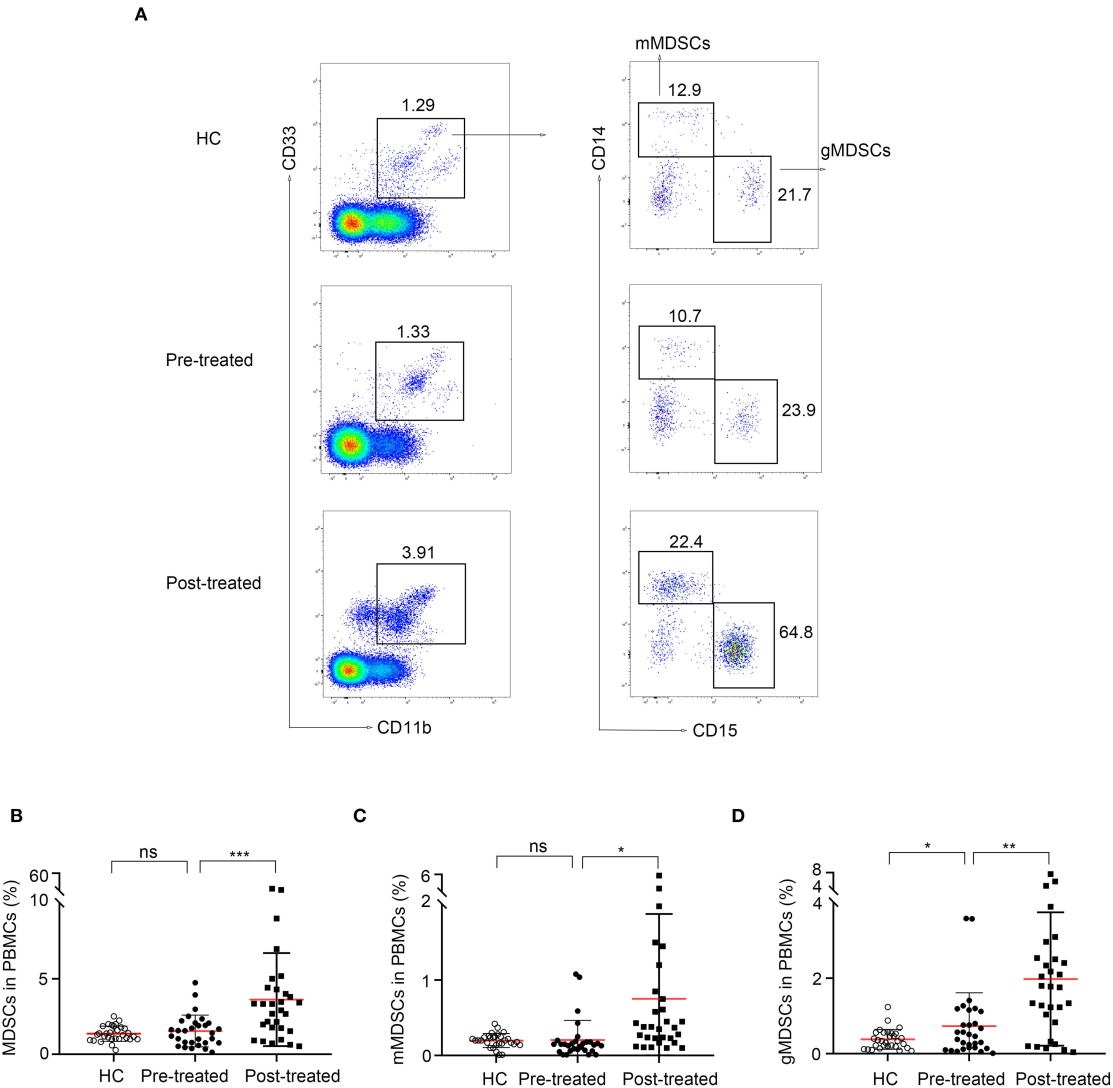
Expansion of MDSCs in patients with myasthenia gravis (MG) after immunotherapy. **(A)** Representative data plots of MDSCs, mMDSCs (HLA-DR^−/low^CD33^+^CD11b^+^CD14^+^) and gMDSCs (HLA-DR^−/low^CD33^+^CD11b^+^CD15^+^) from healthy controls (HC), patients with MG before (pre-treated) and after immunotherapy (post-treated). No difference of frequencies of MDSC **(B)** and mMDSCs **(C)** in PBMC between untreated patients with MG and HCs, but an increased frequency of gMDSC **(D)** in untreated patients with MG. The frequency of MDSCs **(B)**, both mMDSCs **(C)** and gMDSCs **(D)** was significantly increased after immunotherapy. Horizontal lines and error bars represent mean ± SD, *n* = 30, *p < 0.05, **p < 0.01.

Representative data plots of MDSCs, mMDSCs (CD14^+^CD15^−^) and gMDSCs (CD14^−^CD15^+^) from HC, patients before and after immunotherapy were shown in Figure 2A. The frequencies of MDSC and CD14^+^CD15^−^ mMDSCs in PBMC from 30 untreated patients with MG did not significantly change compared to those in 30 HCs (1.5 ± 1.1% vs. 1.4 ± 0.5%, *p* = 0.061, Figure 2B and 0.21 ± 0.26% vs. 0.2 ± 0.094, *p* = 0.85, Figure 2C, respectively). However, the frequency of CD14^−^CD15^+^ gMDSCs increased (0.73 ± 0.88% vs. 0.38 ± 0.26%, *p* = 0.04, Figure 2D) comparing with that in HCs.

Then, we compared the frequency of MDSCs in patients with MG before and after immunotherapy. The frequency of MDSCs (1.5 ± 1.1% vs. 3.6 ± 3.1%, *p* = 0.0009, Figure 2B), both CD14^+^ mMDSCs (0.21 ± 0.26% vs.0.75 ± 1.1%, *p* = 0.017, Figure 2C) and CD15^+^ gMDSCs (0.73 ± 0.88% vs. 2 ± 1.8%, *p* = 0.0011, Figure 2D) was significantly increased after immunotherapy. The result demonstrated that MDSCs and their subsets are all expanded in patients with MG after immunotherapy.

### Correlation of the Frequency of MDSCs With That of Th1, Th17 Cells, Treg, and Bregs

**Figure 3:**
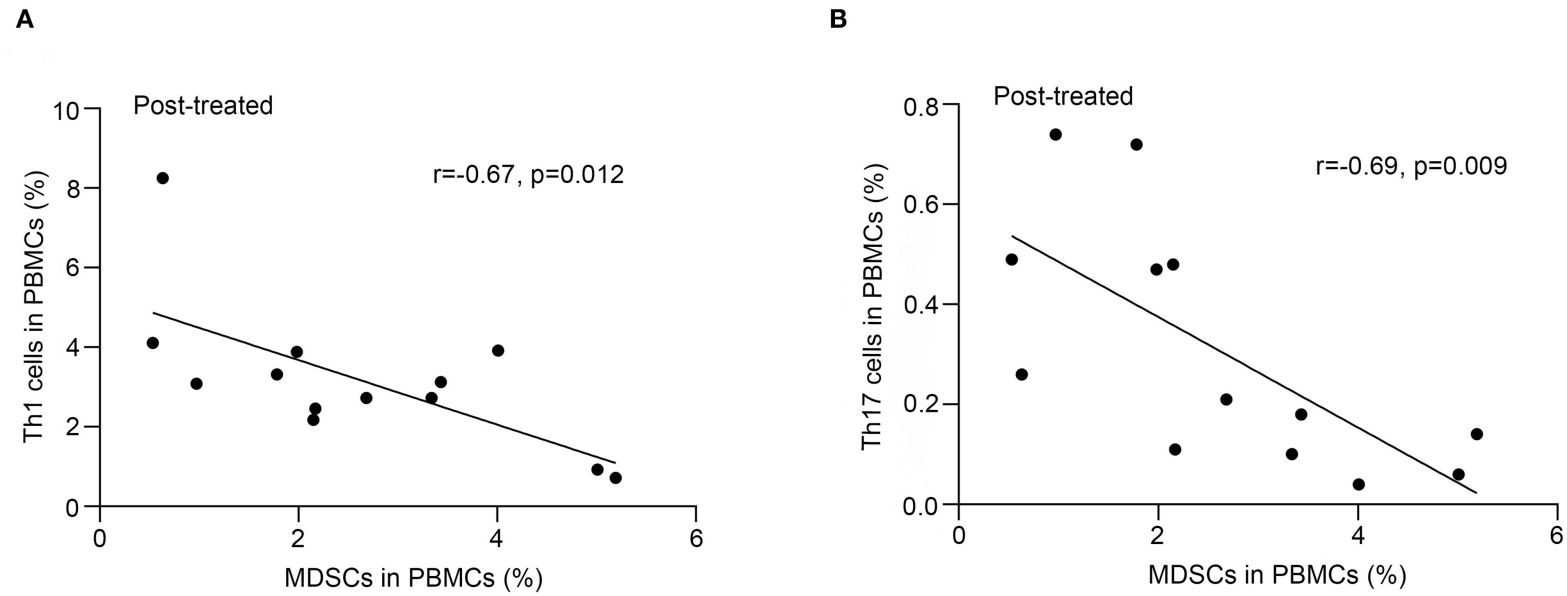
Correlation of frequency of MDSC in PBMCs with that of Th17 and Th1 cells after immunotherapy. Frequency of MDSCs in PBMCs negatively correlated with that of Th1 cells **(A)** or Th17 cells **(B)** in PBMCs from 13 patients after immunotherapy (*n* = 13).

As shown in Figure 3, we observed a negative correlation between the frequency of MDSC and that of Th1 (*r* = −0.67, *p* = 0.012, Figure 3A) or Th17 (*r* = −0.69, *p* = 0.009, Figure 3B) cells in patients with MG after immunotherapy but not in untreated patients (*p* > 0.05). We did not find correlations between the frequency of MDSC and that of Tregs or Bregs (*P* > 0.05).

### Defective Inhibition of IFN-γ Production in T-Cells From Patients With MG Before Immunotherapy

**Figure 4:**
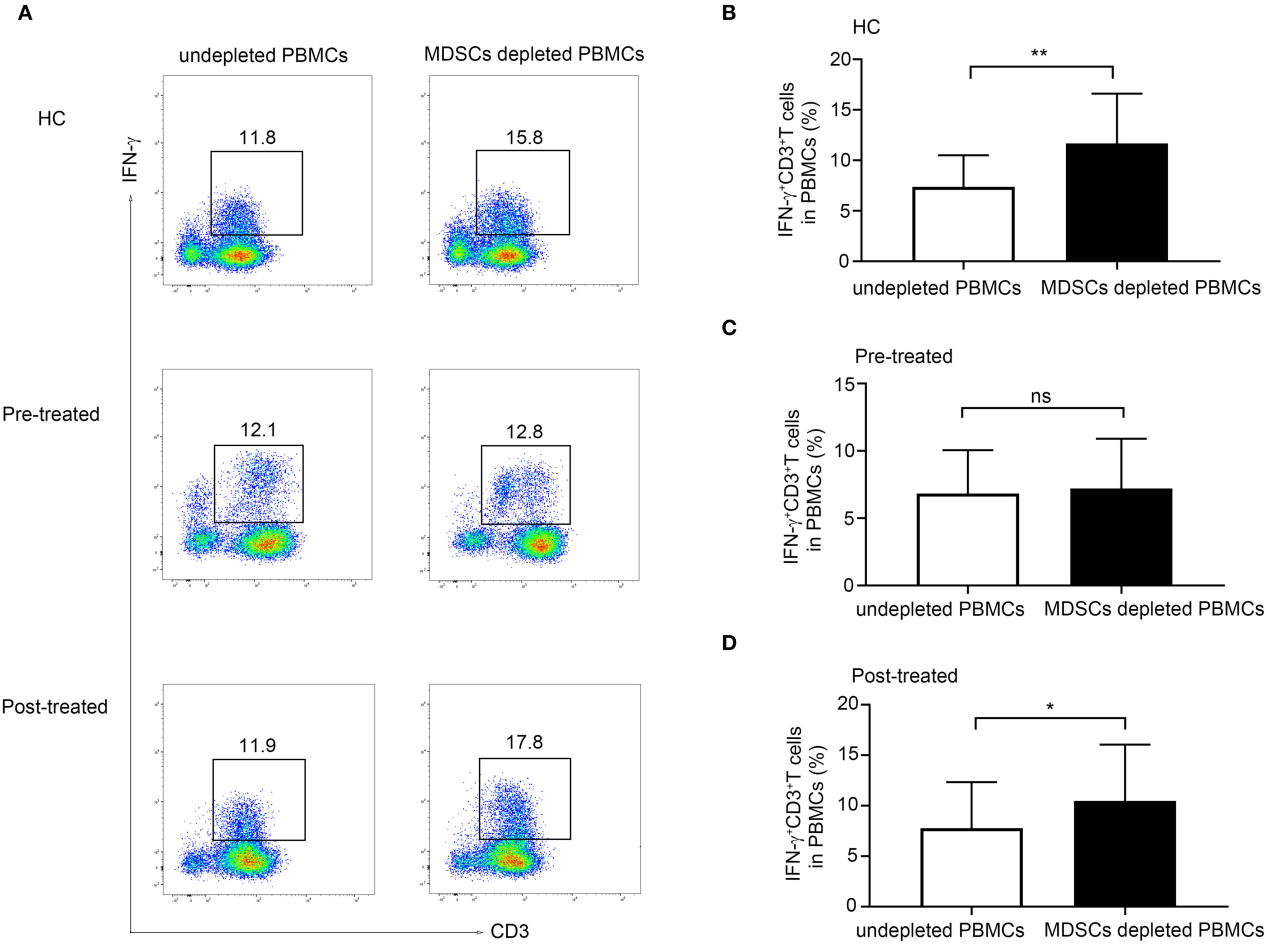
Effect of MDSCs on IFN-γ production in CD3^+^ T-cell from HCs, patients with MG before and after immunotherapy. **(A)** Representative flow cytometry plots of IFN-γ^+^CD3^+^T cells in MDSC depleted PBMCs compared with undepleted PBMCs from HCs, pre-treated, and post-treated patients. A significant increase in the frequency of CD3^+^ IFN-γ^+^ T cells in MDSC depleted PBMCs compared with undepleted PBMCs from both HCs (*n* = 5, **B**) and from patients with MG after immunotherapy (n = 6, **D**), but no significant change in untreated patients with MG (*n* = 6, **C**). Error bars represent mean ± SD. *p < 0.05, **p < 0.01.

As shown in Figure 4A, we observed a significant increase in the frequency of CD3^+^ IFN-γ^+^ T cells in MDSC depleted PBMCs compared with undepleted PBMCs from both HCs (*n* = 5, *p* = 0.0099, Figure 4B) and from patients with MG after immunotherapy (*n* = 6, *p* = 0.033, Figure 4D). However, the depletion of MDSCs from the PBMCs in untreated patients with MG did not lead to an increase in the frequency of CD3^+^IFNγ^+^T cells in PBMCs (*n* = 6, *p* = 0.695, Figure 4C). These findings suggested an impaired suppressive function in untreated patients with MG, and this defect could be ameliorated after immunotherapy.

### Elevated Arg-1 Expression in MDSC After Immunotherapy

Several factors including Arg-1, iNOS, IL-10, PD-1, PD-L1, IL-10, TGF-β, p47phox, gp91phox, and IDO have been implicated in mMDSCs-mediated immunosuppression (9–11, 15–17).

**Figure 5:**
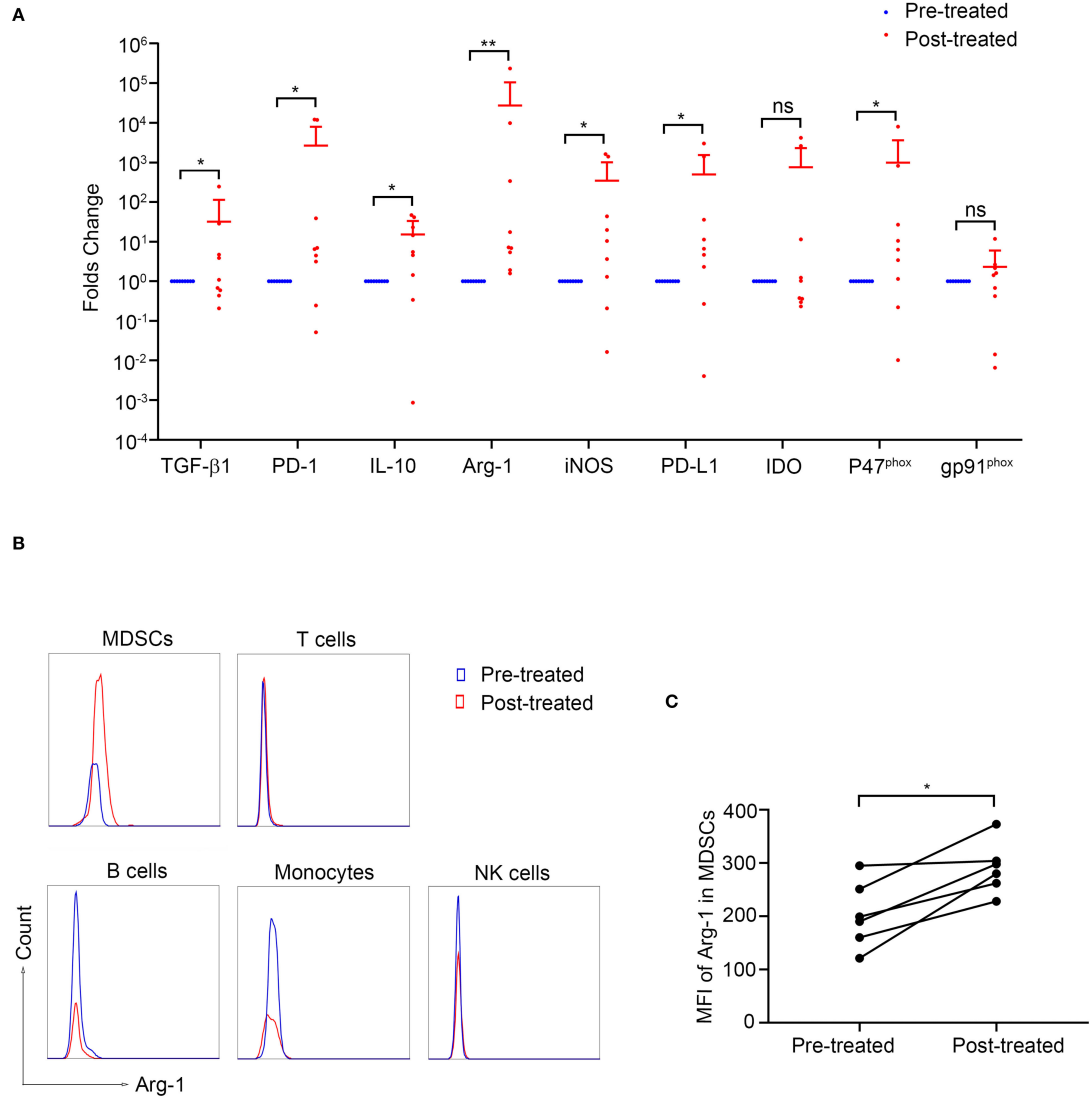
Arginase-1 expression in patients with MG before and after immunotherapy. **(A)** The expression of MDSC-related molecules was measured by qPCR (*n* = 9). *p < 0.05, **p < 0.01. **(B)** Representative flow cytometry analysis of Arg-1 MFI in PBMC subpopulations from pre-treated and post-treated patients. Arg-1 MFI in MDSCs increased after immunotherapy (*n* = 6, **C**). *p < 0.05.

To investigate the potential mechanism by which MDSCs suppress T-cell responses in MG, we measured the intracellular mRNA levels of those factors by quantitative real-time PCR. The expression of TGF-β, iNOS, IL-10, PD-L1, PD-1, and p47phox was upregulated after immunotherapy compared to baseline (*P* < 0.05, Figure 5A) while that of IDO and gp91phox showed no significant difference.

Since the increase of Arg-1 expression identified by qPCR after immunotherapy was most significant (*p* < 0.01, Figure 5A), we next evaluated Arg-1 expression in various cell types by intracellular staining in six patients before and after immunotherapy. As shown in Figure 5B, the intracellular MFI of Arg-1 expression in MDSC was significantly increased after immunotherapy (*p* = 0.031, Figure 5C) while that in T-cells, B-cells, NK-cells, and monocytes showed no statistical differences (*p* > 0.05, Figure S1). These results suggested that the increased expression of Arg-1 identified by qPCR was MDSCs derived.

## Discussion

Myeloid-derived suppressor cells, first identified as immunosuppressive cells, expand in tumor tissues and peripheral blood in patients with malignancies. Considering the role of MDSCs in promoting tumor progression by stimulating tumor cell invasion, angiogenesis, and premetastatic niche development (18), we hypothesized that MDSCs might also play a role in autoimmune diseases and the expansion of MDSCs might alleviate disease severity. However, current knowledge on the role of MDSCs in different human autoimmune diseases is limited and conflicting, possibly due to the instability of MDSCs *in vivo*. In this study, we investigated the phenotypic and functional alterations of MDSC in MG before and after immunotherapy.

Myeloid-derived suppressor cell accumulation has been described in various autoimmune diseases, including rheumatoid arthritis, systemic lupus erythematosus (SLE), inflammatory bowel disease, and multiple sclerosis (19). However, we only identified an increased frequency of gMDSCs but no change of frequencies of MDSCs from untreated patients with MG compared to that from HCs. In agreement with our study, a recent study in relapsing-remitting multiple sclerosis (RR-MS) patients during relapse revealed similar results (20). These results might indicate a different role of mMDSC and gMDSC in autoimmune diseases.

Since there was no quantitative difference between HCs and patients with MG, we suspected function alteration in MDSCs from untreated patients with MG. In a previous study, MDSCs-treated EAMG mice showed significantly suppressed AChR-specific Th1 response compared with control, as measured by IFN-γ production (21). Here, we also explored the effect of MDSCs on T-cell function by evaluating their inhibiting capacity of IFN-γ production in T-cells. Depleting MDSCs in PBMC led to a significant increase of IFN-γ production in T-cells from HCs, but not from untreated patients with MG, which indicated a defect in the suppressive function of MDSCs from untreated MG.

The dynamic quantitive and functional change of MDSCs in different stages of autoimmune diseases has been previously reported. The circulating frequency of CD14^+^HLA-DR^−/low^ MDSCs was significantly increased in newly diagnosed patients with SLE and decreased after immunotherapy (22). RR-MS patients with stable disease stage showed decreased frequencies of CD14^+^HLA-DR^low^ monocytic MDSCs and CD15^+^CD11b^+^HLA-DR^low^ granulocytic MDSCs compared to those during relapse (23). However, our study revealed a significant expansion of MDSCs, including both mMDSCs and gMDSCs after immunotherapy, and the expansion was in line with clinical improvement. We found that seven patients showed no increase in mMDSC or gMDSCs after immunotherapy, and the MG-ADL of these patients was zero or one. There might be a difference between patients who began to improve and those who reached the status of minimal symptom expression. Also, the depletion of MDSCs in PBMC from patients with MG after immunotherapy led to a significant increase in IFN-γ production in T-cells, suggesting an improved inhibiting capacity of MDSCs after immunotherapy.

Oral prednisone and prednisolone are first-line immunotherapy drugs for MG owing to the rapid effect. All the patients were treated with prednisone with a maximum dosage ranging from 20–80 mg or combined with non-steroid immunosuppressants in our cohort. Numerous clinical and experimental data have demonstrated that steroids can enhance the expansion and immunosuppressive function of MDSCs through several mechanisms, including inhibition of HIF1α-dependent glycolysis (24), upregulating the glucocorticoid receptor signaling (25), elevated expression of S100A8/9 (20) and transcription factor Ets1 (26). Furthermore, non-steroid immunosuppressants, tacrolimus, could also modulate the expansion and function of MDSCs (27, 28). The expansion and improved immunosuppressive function of MDSCs in MG after immunotherapy might be due to the effect of steroids and other immunosuppressants. Further longitudinal studies with longer follow-up and *in vitro* experiments are needed to clarify this phenomenon.

Increasing studies have analyzed the association of MDSCs and proinflammatory and regulatory T-cells in human autoimmune diseases. Mainly from studies of SLE and RA, two perspectives exist. Some studies have shown that MDSCs are highly potent in promoting Th17 cell differentiation and disease progression. In contrast, some evidence indicates the opposite effects of MDSCs on decreasing Th17 and Th1 differentiation as well as inducing Foxp3^+^ regulatory T-cell differentiation, which is correlated with a relative improvement in the disease (29–33). MG is a B-cell-mediated, T-cell-dependent autoimmune disease (34). The secretion of IFN-γ and IL-17 in AChR peptide-stimulated CD4^+^ T cells demonstrated that Th1 and Th17 cells are involved in the pathogenesis of MG (35, 36). The association between MDSCs and proinflammatory T cell populations has not been investigated. We observed a negative correlation between the frequency of MDSC and that of Th1 or Th17 cells in patients with MG after immunotherapy but not in untreated patients. The results might arise from the heterogeneous of MDSCs before and after immunotherapy, which had different effects on T-cell proliferation and differentiation. MDSCs could also induce expansion of regulatory B-cells in a murine model of SLE and collagen-induced arthritis (37, 38), but in our study of patients with MG, we found no correlations between the frequency of MDSC and that of Bregs. Accurate elucidation still needs to be further explored *in vitro* experiments and animal models.

It is well known that the depletion of arginine through the upregulation of Arg-1 was one of the first and foremost T-cell suppressive mechanisms described in MDSCs. Arg-1 reduces the nutrient of T-lymphocytes and produces ornithine and polyamines (7, 39). Low levels of L-arginine reduce the zeta chain expression of the TCR-CD3 on T cells, dampen the cell cycling in the G0/G1 phase (inhibiting T cell proliferation) and decrease proinflammatory cytokine production (40, 41). Plasma concentration of Arg-1 was also negatively correlated to the frequency of Th17 cells in RA (32) and disease activity in Sjögren syndrome (42). However, MDSCs from SLE patients exhibited significantly elevated Arg-1 production and increased potential to promote Th17 differentiation *in vitro* in an Arg-1–dependent manner. These studies suggest that the role of Arg-1 in the pathogenesis of autoimmune diseases is not univocal. In MG, our study showed that the expression of MDSC-derived Arg-1 was significantly upregulated after immunotherapy, and the expression of Arg-1 in MDSCs was negatively correlated with disease severity measured by MG-ADL. Further studies are required to explore the subtle role of Arg-1 for T cell proliferation and Th17 differentiation in MG.

There are some limitations in our study: (1) The patients were followed only for 6–12 months, and the long-term phenotypic and quantitive changes of MDSCs are still unknown; (2) Lacking the direct study of a suppressive effect of MDSCs on T-cell proliferation (3) Further *in vitro* and *in vivo* studies are needed to elucidate the role of MDSC derived Arg-1 in T-cell suppression and differentiation; (4) Since difficulties obtaining sufficient blood samples from all patients with MG, a limited sample size for correlated analysis between frequency of MDSCs and Th17/Th1, semi-quantity analysis of Arg-1 expression and IFN-γ production in T cells with and without MDSCs.

Although no frequency difference compared to HCs, MDSCs significantly expanded, and the frequency of MDSCs negatively correlated to that of Th1 or Th17 cells after immunotherapy. MDSCs from untreated patients with MG showed an impaired suppression of IFN-γ production in T cells and improved immunosuppressive function was identified after immunotherapy. The MFI of Arg-1 in MDSCs also increased after immunotherapy. These findings suggested the functional difference in MDSCs before and after immunotherapy, and MDSCs might play a role in disease remission.

## Data Availability Statement

The raw data supporting the conclusions of this article will be made available by the authors, without undue reservation.

## Ethics Statement

The studies involving human participants were reviewed and approved by the Institutional Review Board of Huashan Hospital, Fudan University. The patients/participants provided their written informed consent to participate in this study.

## Author Contributions

YaW designed the study, generated and analyzed the experimental data, and drafted the original manuscript. CY reviewed, edited, and approved the final version of the manuscript. CS, YiW, SL, and JL collected clinical data. CZ critically reviewed, edited, and approved the final version of the manuscript. GZ assisted in conceptualizing and designing the study, interpreted the experimental data, and drafted part of the original manuscript. JX conceptualized the study, interpreted the experimental and clinical data comprehensively, critically reviewed, edited, and approved the final version of the manuscript. All authors contributed to the article and approved the submitted version.

## Funding

This research was supported by the National Key Research and Development Program of China (No.2017YFD0502301-2) and the National Natural Science Foundation of China (No. 81901279 and 81803542).

## Conflict of Interest

The authors declare that the research was conducted in the absence of any commercial or financial relationships that could be construed as a potential conflict of interest.

## Publisher's Note

All claims expressed in this article are solely those of the authors and do not necessarily represent those of their affiliated organizations, or those of the publisher, the editors and the reviewers. Any product that may be evaluated in this article, or claim that may be made by its manufacturer, is not guaranteed or endorsed by the publisher.

## Abbreviations

AChR: acetylcholine receptor
Arg-1: Arginase-1
gMDSC: granulocytic MDSC
HC: healthy controls
IFN-γ: interferon γ
IL: interleukin
IDO: indoleamine-2 3-dioxynase
iNOS: inducible nitric oxide synthase
mMDSC: monocytic MDSC
MDSC: myeloid-derived suppressor cell
MG: myasthenia gravis
PBMC: peripheral blood mononuclear cell
PE: phycoerythrin
PerCP: peridinin chlorophyll protein
PMA: phorbol 12-myristate 13-acetate
ROS: reactive oxygen species
TGF-β: transforming growth factor-β.
