# Supplementary material for: Increased Frequency of Myeloid-Derived Suppressor Cells in Myasthenia Gravis After Immunotherapy
Source: Front Neurol. 2022 Jun 29;13:902384. doi: 10.3389/fneur.2022.902384 (PMC9278661; doi:10.3389/fneur.2022.902384)
Supplement: Supplementary file 1 [file Table_1.DOCX]

**Table S1** Primers used for qPCR in this study

| **Gene** | **Forward Primers (5’-3’)** | **Reverse Primers (5’-3’)** | **Product length(bp)** |
| --- | --- | --- | --- |
| Arg-1 | GTT TTG ATG TTG ACG GAC TG | GTA GCC CTG TTT TGT AGA TT | 120 |
| iNOS | GGT GAA AGA TGG AAC TTG CT | AAA TAC ACA GTG GTG CGA TA | 77 |
| TGFβ1 | ACC TGA ACC CGT GTT GCT CT | GAA CCC GTT GAT GTC CAC TT | 286 |
| IL-10 | CCA AGA GAA AGG CAT CTA CA | GGG GGT TGA GGT ATC AGA G | 84 |
| PD-L1 | GGC TGA GCA AGG CAC ATA GT | CAC CAC AAG GAG GAG TTA GG | 61 |
| PD-1 | GGA AAC CCC TCC ACC TTT AC | TCT GCC TGC CCG CTT ACT | 88 |
| IDO | CCC TTC AAG TGT TTC ACC AA | GTC TTC CCA GAA CCC TTC AT | 137 |
| gp91 | TAT GAG GTG GTG ATG TTA GT | TTC AGA TTG GTG GCG TTA TT | 104 |
| P47phox | GCT GTT GAG GTC ATT CAC AA | GCT GTT GAG GTC ATT CAC AA | 80 |
| β-actin | AAG GTG ACA GCA GTC GGT T | TGT GTG GAC TTG GGA GAG G | 195 |

| **No** | **Sex** | **Age(y)** | **Diseseas duration (m)** | **Thymus^*^** | **AChR Ab titer (nmol/L）** | | **MGFA classification** | | **QMG score** | | **MG-ADL** | | **Treatment^#^** |
| --- | --- | --- | --- | --- | --- | --- | --- | --- | --- | --- | --- | --- | --- |
|  |  |  |  |  | **Pre-treat** | **Post-treat** | **Pre-treat** | **Post-treat** | **Pre-treat** | **Post-treat** | **Pre-treat** | **Post-treat** |  |
| 1 | F | 72 | 3 | (-) | 8.11 | 3.36 | 2a | 2a | 12 | 7 | 6 | 3 | P 30mg, AZA 75mg |
| 2 | M | 33 | 29 | (-) | 15.17 | 15.21 | 2a | 2a | 8 | 3 | 1 | 0 | P 30mg |
| 3 | F | 54 | 1 | (-) | 1.84 | 0.255 | 4b | MMS | 11 | 1 | 5 | 0 | P 40mg |
| 4 | M | 62 | 15 | (-) | 19.51 | 16.66 | 2a | 1 | 10 | 5 | 4 | 1 | P 50mg, MMF 1500mg |
| 5 | M | 20 | 2 | (-) | 11.13 | 9.97 | 2a | MMS | 7 | 0 | 2 | 0 | P 30mg |
| 6 | F | 74 | 1 | (-) | 14.32 | 4.47 | 2b | 2a | 11 | 6 | 8 | 3 | P 54mg |
| 7 | M | 29 | 8 | (-) | positive | NA | 3a | 2a | 19 | 7 | 9 | 1 | P 20mg |
| 8 | F | 57 | 1 | AB | 9.18 | 3.69 | 2b | 2a | 19 | 13 | 12 | 4 | Tx, P 30mg |
| 9 | F | 31 | 17 | (-) | 15.7 | 15.42 | 4a | 2a | 23 | 4 | 12 | 0 | P 20mg |
| 10 | F | 71 | 1 | Thymoma,  not Tx | 15.95 | 1.94 | 3a | 2a | 12 | 5 | 7 | 3 | P 30mg, TAC 3mg |
| 11 | M | 42 | 11 | (-) | 9.31 | NA | 4a | 2a | 28 | 5 | 14 | 2 | P 35mg |
| 12 | F | 67 | 7 | (-) | 11.56 | 5.32 | 3a | 2a | 20 | 10 | 9 | 3 | P 30mg, AZA 100mg |
| 13 | M | 19 | 1 | (-) | 1.64 | NA | 3a | 2a | 15 | 11 | 8 | 4 | P 50mg, TAC 3mg |
| 14 | M | 41 | 1 | (-) | 8.05 | 7.68 | 2b | MMS | 6 | 1 | 4 | 0 | P 30mg |
| 15 | F | 50 | 7 | B2 | 4.64 | NA | 3b | 2b | 16 | 10 | 12 | 3 | Tx, M 24mg |
| 16 | M | 73 | 36 | (-) | 19.49 | 14.58 | 2a | 2a | 12 | 8 | 12 | 8 | P 35mg |
| 17 | F | 28 | 24 | (-) | 1.98 | 1.53 | 3b | 2b | 12 | 9 | 6 | 2 | P 30mg |
| 18 | M | 54 | 2 | B1 | 12.83 | 8.05 | 2b | 1 | 8 | 1 | 6 | 1 | P 20mg |
| 19 | M | 43 | 3 | (-) | 3.5 | 1.05 | 2a | 2a | 11 | 6 | 19 | 2 | P 30mg |
| 20 | F | 37 | 13 | B2 | 12.83 | 12.01 | 2a | 2a | 6 | 3 | 2 | 0 | P 20mg |

**Tables S2** Clinical details of all enrolled patients

| **No** | **Sex** | **Age(y)** | **Diseseas duration (m)** | **Thymus^*^** | **AChR Ab titer (nmol/L)** | | **MGFA classification** | | **QMG score** | | **MG-ADL** | | **Treatment^#^** |
| --- | --- | --- | --- | --- | --- | --- | --- | --- | --- | --- | --- | --- | --- |
|  |  |  |  |  | **Pre-treat** | **Post-treat** | **Pre-treat** | **Post-treat** | **Pre-treat** | **Post-treat** | **Pre-treat** | **Post-treat** |  |
| 21 | M | 73 | 36 | (-) | 11.62 | 8.39 | 2b | 2a | 13 | 4 | 8 | 3 | P 20mg |
| 22 | F | 46 | 5 | B3 | 12.3 | 16.12 | 3a | 3a | 16 | 8 | 7 | 0 | P 40mg |
| 23 | M | 62 | 1 | AB | 13.29 | NA | 2b | 2a | 7 | 5 | 5 | 3 | M 80mg, IVIG |
| 24 | M | 65 | 2 | (-) | 9.61 | 3.29 | 2b | MMS | 8 | 0 | 5 | 0 | P 30mg |
| 25 | F | 69 | 1 | (-) | 8.17 | 5.33 | 3b | 2b | 12 | 4 | 8 | 2 | P 30mg, MMF 750mg, IVIG |
| 26 | F | 62 | 200 | (-) | 6.49 | 6.93 | 3a | 2a | 15 | 9 | 7 | 2 | P 30mg |
| 27 | F | 22 | 15 | (-) | 14.54 | 14.02 | 3a | 2a | 12 | 4 | 4 | 1 | Tx, P 20mg, TAC 3mg |
| 28 | F | 78 | 7 | (-) | 11.84 | 7.28 | 2b | 2a | 12 | 9 | 13 | 3 | Tx, M 80mg |
| 29 | M | 19 | 2 | (-) | 17.63 | NA | 2a | MMS | 12 | 1 | 6 | 0 | P 50mg |
| 30 | F | 19 | 4 | (-) | 5.11 | NA | 3a | 3a | 11 | 8 | 5 | 2 | P 30mg, TAC 3mg |

Abbreviations: Pre-treated = before immunotherapy; Post-treated = after immunotherapy; MGFA = Myasthenia Gravis Foundation of America; AChR = acetylcholine receptor; QMG score = quantitative MG score; MG-ADL = MG Activities of Daily Living; F = female; M = male; NA = not available; MMS = minimal manifestation status; Tx = thymectomy; P = prednisolone; M = methylprednisolone; AZA = Azathioprine; TAC = Tacrolimus; MMF = Mycophenolate Mofetil; IVIG = intravenous immunoglobulin.

*The WHO histologic classification of thymoma for patients with thymectomy. (-) No thymoma was found by CT scan.

^#^Maximum daily dosage of steroid and the total daily dose of nonsteroidal immunosuppressants.

The dosage of steroids was adjusted according to the status of patients. All the patients were given oral steroids with an initial dose of 20mg prednisone every day. The dose was increased by increments of 10 mg every two weeks without exceeding 0.75 mg/kg/d of body weight. The dose was maintained for 1-2 months when showing a ≥2-point reduction in ADL-MG and then reduced by 5 mg every 1-2 months. Intravenous methylprednisolone was administrated in 2 patients during hospitalization. Non-steroid immunosuppressants were added for patients who did not respond to steroids or refused to take a higher dose of oral steroids.


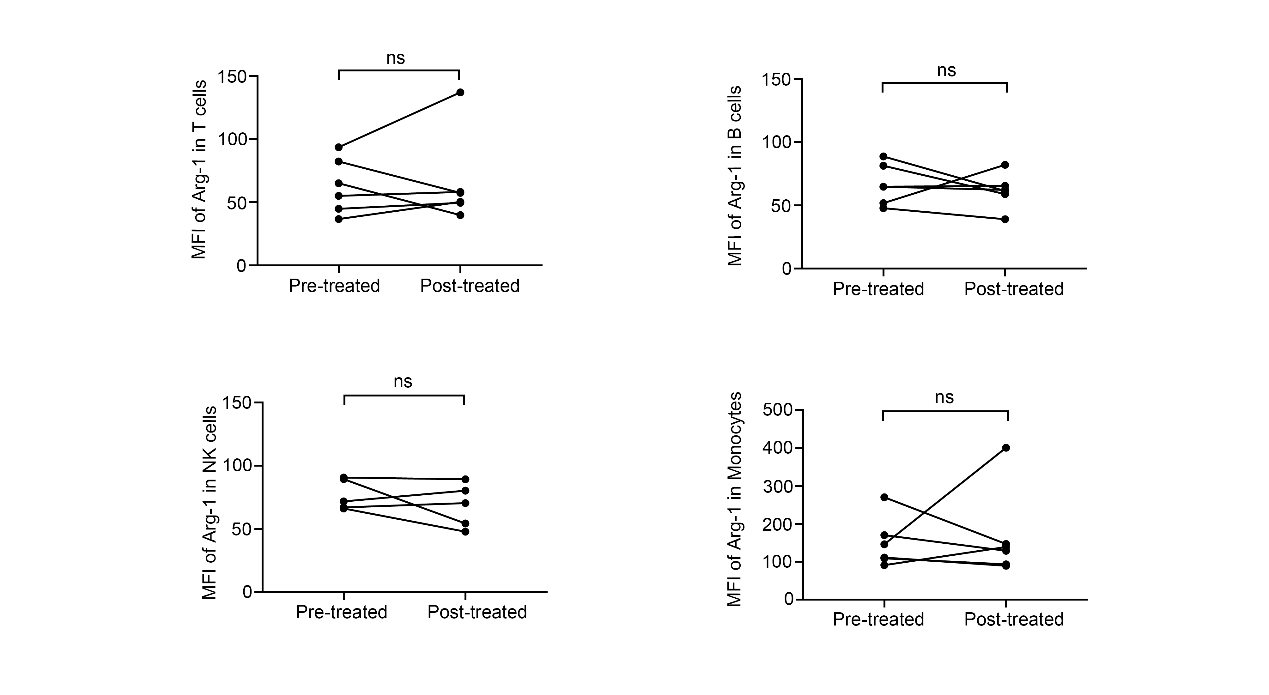


**Figure S1.** MFI of Arginase-1 in T cells (n=6), B cells (n=6), monocytes (n=6) and NK cells (n=5) showed no difference in patients with MG before and after immunotherapy.
